# Microvasculopathy-Related Hemorrhagic Tissue Deposition of Iron May Contribute to Fibrosis in Systemic Sclerosis: Hypothesis-Generating Insights from the Literature and Preliminary Findings

**DOI:** 10.3390/life12030430

**Published:** 2022-03-16

**Authors:** Petros P. Sfikakis, Nikolaos I. Vlachogiannis, Panagiotis A. Ntouros, Sophie Mavrogeni, Thomas G. Maris, Apostolos H. Karantanas, Vassilis L. Souliotis

**Affiliations:** 1 First Department of Propaedeutic Internal Medicine and Joint Academic Rheumatology Program, National and Kapodistrian University of Athens Medical School, 11527 Athens, Greece; nvlachog@med.uoa.gr (N.I.V.); panantou@med.uoa.gr (P.A.N.); vls@eie.gr (V.L.S.); 2 Onassis Cardiac Surgery Center, 17674 Athens, Greece; mavrogeni@ocsc.gr; 3 Department of Radiology, University of Crete Medical School, 71003 Heraklion, Greece; marist@uoc.gr (T.G.M.); karantanas@uoc.gr (A.H.K.); 4 Computational BioMedicine Laboratory, Institute of Computer Science, Foundation for Research and Technology-Hellas (FORTH), 70013 Heraklion, Greece; 5 Institute of Chemical Biology, National Hellenic Research Foundation, 11635 Athens, Greece

**Keywords:** systemic sclerosis, fibrosis, iron deposition, oxidative stress, vasculopathy, MR imaging/quantification

## Abstract

Microvascular wall abnormalities demonstrated by nailfold capillaroscopy in systemic sclerosis (SSc) may result in microhemorrhagic deposition of erythrocyte-derived iron. Such abnormalities precede fibrosis, which is orchestrated by myofibroblasts. Iron induces endothelial-to-mesenchymal transition in vitro, which is reversed by reactive oxygen species (ROS) scavengers. The conversion of quiescent fibroblasts into profibrotic myofibroblasts has also been associated with ROS-mediated activation of TGF-β1. Given that iron overload predisposes to ROS formation, we hypothesized that the uptake of erythrocyte-derived iron by resident cells promotes fibrosis. Firstly, we show that iron induces oxidative stress in skin-derived and synovial fibroblasts in vitro, as well as in blood mononuclear cells ex vivo. The biological relevance of increased oxidative stress was confirmed by showing the concomitant induction of DNA damage in these cell types. Similar results were obtained in vivo, following intravenous iron administration. Secondly, using magnetic resonance imaging we show an increased iron deposition in the fingers of a patient with early SSc and nailfold microhemorrhages. While a systematic magnetic resonance study to examine tissue iron levels in SSc, including internal organs, is underway, herein we propose that iron may be a pathogenetic link between microvasculopathy and fibrosis and an additional mechanism responsible for increased oxidative stress in SSc.

## 1. Introduction

Systemic sclerosis (SSc) is a devastating chronic disease characterized by the unique triad of vasculopathy, (auto)immune activation, and fibrosis [1]. All patients show signs of microvasculopathy, typically expressed as Raynaud’s phenomenon, digital ischemia, and ulcer development [2]. Nailfold capillaroscopy, which has been integrated in the 2013 SSc classification criteria [3], shows that patients with SSc are characterized by multiple microvascular pathologies, such as enlarged capillaries, capillary loss, and hemorrhages [4,5]. Microvascular abnormalities are strong independent predictors of the progression from primary Raynaud’s phenomenon to definite SSc [4], underlining the central pathogenetic role of vasculopathy. Moreover, vascular endothelial cells may become transformed through endothelial-to-mesenchymal transition (EndoMT) to profibrotic myofibroblasts [6], which are thought to orchestrate fibrosis [7,8]. Thus, the fibrotic process of the skin and internal organs, which becomes clinically evident months to years after Raynaud’s onset, is considered to be driven by the chronic vascular injury/repair process and a concomitant (auto)immune activation [9,10].

As depicted in capillaroscopy images, microvascular damage and extravasation of erythrocytes occurs even at the earliest stages of SSc [5]. Such microhemorrhages could lead to erythrocyte-derived iron deposition in surrounding tissues. Iron is an essential element present within the cells mainly in association with hemoproteins (hemoglobin, myoglobin) or within an iron–sulfur cluster of various metalloproteins [11]. Iron is involved in the redox-driven processes of oxygen transport, electron transport, and various enzymatic reactions [12]. Cellular iron uptake and storage is carefully regulated so as to avoid deficiency or excess; excess iron can lead to cell damage by promoting the generation through the Fenton reaction of extremely reactive hydroxyl radicals (^•^OH) [13,14], which can oxidize whatever happens to be in the vicinity of their formation, including DNA, RNA, lipids, and proteins. Importantly, it is not the total amount of iron that is responsible for mediating the oxidative damage, but rather the labile Fe(II) fraction pool that is accessible for interaction with peroxides [15]. The depletion of intracellular labile iron by exogenous compounds would diminish the formation of damaging ROS and prevent the deleterious over-oxidation of cellular components [16]. Several lines of evidence suggest that ROS-induced oxidization of lipids and proteins promotes initiation and progression of fibrosis in a variety of organs [17,18,19,20]. Similarly, in vitro experiments and experiments in animal models have shown that iron may enhance TGF-b production by mesenchymal cells [21], induce fibroblast-to-myofibroblast transition associated with increased collagen production [22,23], induce EndoMT [24], and promote vascular smooth muscle cell proliferation [25], mainly through increased oxidative damage and ROS formation.

Based on the above we formulated the hypothesis that hemorrhagic tissue deposition of iron, due to microvasculopathy-related extravasation of erythrocytes, may be of pathogenetic importance in fibrosis in SSc, thus comprising an additional therapeutic target. To support this hypothesis, herein, we describe the induction of oxidative stress and the concomitant accumulation of DNA damage in human fibroblasts in vitro, and in peripheral blood mononuclear cells (PBMCs) ex vivo after treatment with iron, as well as in vivo after therapeutic intravenous iron administration. Moreover, we provide preliminary results of increased iron deposition in the hands in early SSc, as shown with magnetic resonance imaging (MRI).

## 2. Materials and Methods

### 2.1. Cell Lines and PBMCs

Human SW-982 cells (fibroblast-like synoviocytes) and human HDF-a cells (dermal fibroblasts-adult; Thermo Fisher Scientific, Waltham, MA, USA) were maintained in Dulbecco’s Modified Eagle Medium (DMEM), supplemented with 10% fetal bovine serum (FBS) and 1% penicillin-streptomycin in a humidified atmosphere with 5% CO_2_ at 37 °C.

Peripheral blood was collected from 3 individuals with iron deficiency anemia before and immediately after their first therapeutic intravenous iron (ferric carboxymaltose, Ferrinject, Vifor, France) administration over 2 h (500 mg Fe(III) diluted in 250 mL of normal saline), and from 3 healthy controls without iron treatment. PBMCs were isolated using the Ficoll gradient centrifugation, as previously described [26]. All individuals involved in the study gave their informed consent according to the declaration of Helsinki. The study was approved by Laiko Hospital Ethics Committee (Protocol Nr.1110/22.01.2021).

### 2.2. Chemicals and Cell Treatment

To prepare a stock solution with a final iron concentration of 10 mM, ferric nitrate (40 mg) (Fe(NO_3_)_3_; Sigma-Aldrich, #254223, St. Louis, MO, USA) was dissolved in 10 mL of distilled water and the chelating agent disodium nitrilotriacetate (23.5 mg) (NTA; Sigma-Aldrich, #N0128) was added. The pH of the solution was adjusted to 7.4 by sodium hydrogen carbonate (NaHCO_3_; Sigma-Aldrich, #S8875) [27]. All chemicals were of analytical grade.

PBMCs were treated with 10 and 100 μM of freshly prepared FeNTA for 30 min. in a complete RPMI-1640 medium supplemented with 10% FBS, 100 units/mL penicillin, 100 mg/mL streptomycin, and 2 mmol/L L-glutamine. Synovial and dermal fibroblast cell lines were also treated with 10/100 μM and 100 μM FeNTA for 30 min. at 37 °C in culture medium, respectively. Cells were resuspended in freezing medium (90% FBS, 10% dimethyl sulfoxide) and stored at −80 °C until further processing. All experiments were performed at least three times.

### 2.3. Measurement of Oxidative Stress

Oxidative stress was measured using a luminescence-based system that detects and quantifies total glutathione (GSH), oxidized glutathione (glutathione disulfide (GSSG)), and the GSH/GSSG ratio according to the manufacturer’s protocol (GSH/GSSG-Glo^TM^ Assay, Promega, Madison, WI, USA).

### 2.4. Measurement of DNA Damage

DNA damage levels were measured by single cell gel electrophoresis (comet assay) under alkaline conditions, measuring single-strand breaks (SSBs) and/or double-strand breaks (DSBs) as previously described [28]. Abasic (apurinic/apyrimidinic) sites were evaluated using the OxiSelect™ Oxidative DNA Damage Quantitation Kit (AP Sites; Cell Biolabs, #STA-324) according to the manufacturer’s protocol.

### 2.5. Magnetic Resonance Imaging Protocol of the Hands

Examination was performed on a 3T superconducting clinical MR imager (SKYRA system, Siemens, Erlangen, Germany), (gradient strength: 45 mT·m^−1^, gradient rise time: 300 μs, gradient slew rate: 200 mT·m^−1^·s^−1^). A system-embedded RF body coil was used for signal excitation and a standard 20-channel phased array head/neck coil was used for signal detection.

For the quantitation of iron deposition, a quantitative T2STAR imaging sequence was utilized. The final T2STAR quantitative imaging protocol consisted of a gradient echo sequence which was utilized under a multi-echo-gradient-echo (MEGRE) train scheme using 8 symmetrically repeated gradient echoes. The first TE was 1.62 ms and the other 7 TEs were obtained thereafter every 2.43 ms, that is, the TEs were: 4.05, 6.48, 8.91, 11.34, 13.77, 16.2, and 18.63 ms. With these chosen TE values, a sensitive multiecho sequence for single exponential T2STAR measurements ranging from 2 ms up to 90 ms was obtained. A standard TR of 700 ms was used. The relative MEGRE sequence contrast-related parameters were therefore: (TR/TE1.../TE8/FA: 700 ms/1.62 ms…/18.63 ms/20°). One signal average and a receiver bandwidth of 500 Hz/pixel were used. The total examination time was approximately 16 s. One coronal slice of 8 mm slice thickness was used. A field of view (FOV) of 270 × 360 mm^2^ was utilized with a 192 × 256 matrix The final pixel dimensions (FOV/MTX) therefore corresponded to a square pixel matrix with pixel dimensions 1.4 × 1.4 mm^2^ (in-plane spatial resolution). The overall spatial resolution expressed in raw data voxel dimensions was 1.4 × 1.4 × 6 mm^3^. The longer anatomical axis (head to feet direction for the coronal slices) was always chosen as the frequency-encoding axis. The highest possible receiver bandwidth (500 Hz/pixel) was used in order to eliminate geometric distortions due to susceptibility artifacts. A 2D geometric distortion filtering was also applied in order to eliminate geometric distortions due to inherent gradient field imperfections. Both hands were imaged simultaneously. T2STAR values were calculated on a pixel-by-pixel basis and finally, T2STAR color parametric maps were produced directly from the clinical system embedded software program.

### 2.6. Statistics

The normality of the data distribution was controlled by the Shapiro–Wilk test. Differences in continuous variables were examined by an independent samples *t*-test or a Mann–Whitney U test when non-normally distributed. Differences in paired measurements were examined by a paired samples *t*-test or the nonparametric Wilcoxon’s signed rank test. Results were considered statistically significant when P < 0.05.

## 3. Results

### 3.1. Iron Overload Induces Oxidative Stress in Fibroblasts and PBMCs In Vitro

**Figure 1:**
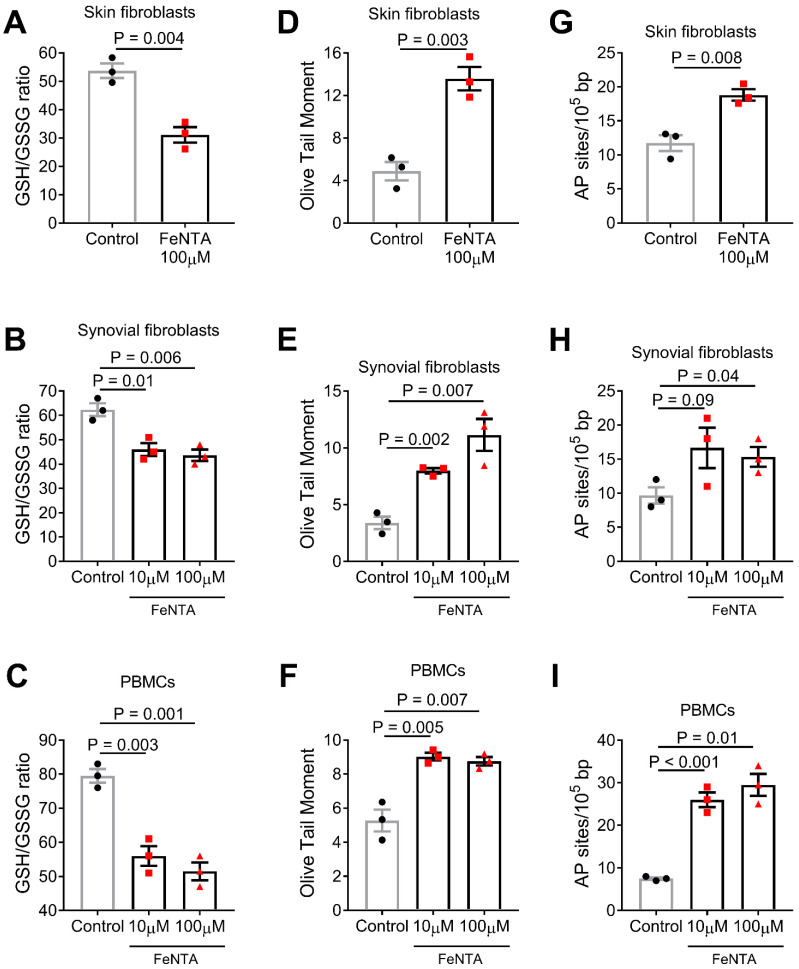
**Iron-induced oxidative stress and DNA damage in fibroblasts in vitro and PBMCs ex vivo.** Bar graphs shows oxidative stress levels expressed as the GSH/GSSG ratio (**A**–**C**), DNA damage expressed as Olive Tail Moment arbitrary units (**D**–**F**) or AP sites (**G**–**I**) after in vitro treatment with 10 and 100 μM FeNTA. P-values are derived from independent samples *t*-test (or Welch’s *t*-test when there were unequal variances).

The Fenton reaction has been much studied as a source of hydroxyl radicals and initiator of biological damage [15]. Thus, herein, we firstly examined the effect of iron excess on the oxidative stress status of human-skin-derived (HDF-a) and synovial-derived (SW-982) fibroblasts. We found that following treatment of these cell lines with 100 μM FeNTA for 30 min, FeNTA-treated cells showed significantly higher levels of oxidative stress (as indicated by the reduction of the GSH/GSSG ratio) compared with the untreated cells (HDF-a: P = 0.004, Figure 1A; SW-982: P = 0.006, Figure 1B). Similar results were observed in PBMCs from the three healthy controls after ex vivo treatment with FeNTA (P = 0.001, Figure 1C). Since normal serum iron reference ranges between 10–35 μM depending on age, sex, and other conditions, we also performed experiments using 10 μM FeNTA for 30 min. Comparable results were obtained in both synovial-derived fibroblasts and PBMCs (Figure 1B,C), suggesting that there is no dose-dependent effect within the 10–100 μM range.

It is well-known that increased levels of ROS can result in various types of DNA damage, including SSBs, DSBs, and abasic sites. Therefore, the iron-induced oxidative DNA damage in the fibroblast cell lines and PBMCs mentioned above was analyzed using alkaline comet assay measuring SSBs and/or DSBs. We found that FeNTA-treated fibroblasts and PBMCs showed significantly higher levels of DNA damage compared with nontreated cells (HDF-a: P = 0.003, Figure 1D; SW-982: P = 0.007, Figure 1E; PBMCs: P = 0.007, Figure 1F). Similar results were obtained in the measurement of abasic sites (HDF-a: P = 0.008, Figure 1G; SW-982: P = 0.04, Figure 1H; PBMCs: P = 0.01, Figure 1I). Again, comparable results were obtained regarding DNA damage using 10 μM FeNTA for 30 min. in both synovial-derived fibroblasts and PBMCs, (Figure 1E,F, respectively), as well as regarding abasic sites (Figure 1H,I, respectively).

### 3.2. In Vivo Induction of Oxidative Stress in PBMCs Following Intravenous Administration of Iron

**Figure 2:**
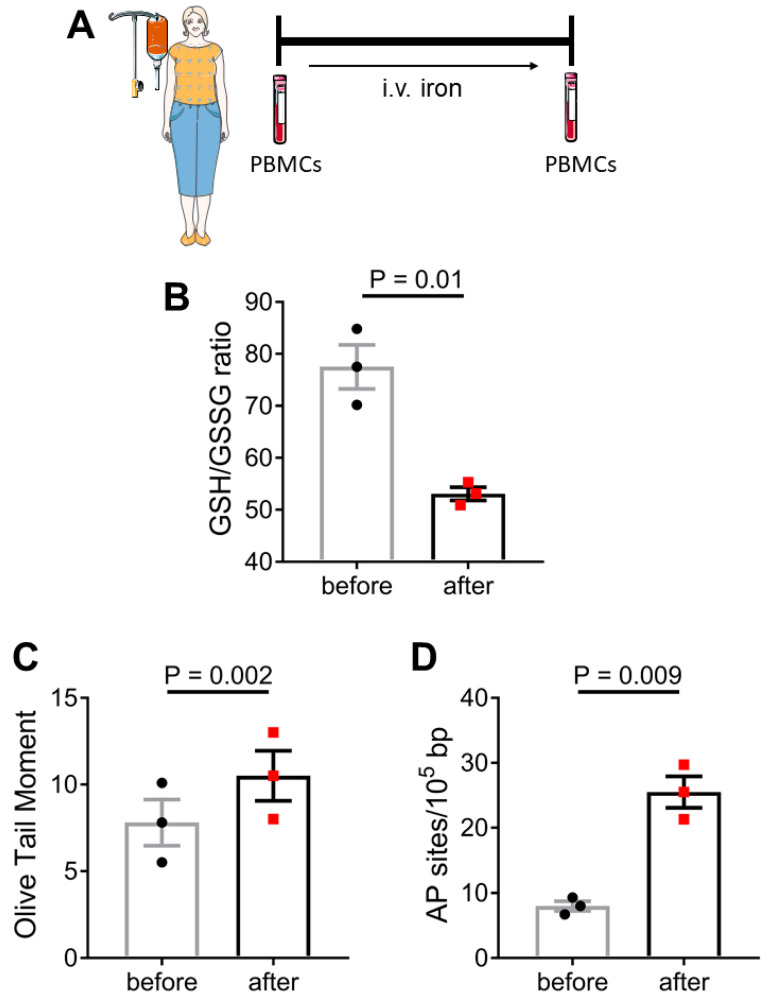
**Iron-induced oxidative stress and DNA damage in PBMCs in vivo.** PBMCs were isolated from 3 individuals with iron deficiency anemia before and immediately after therapeutic intravenous iron administration (**A**). Bar graphs show oxidative stress levels expressed as the GSH/GSSG ratio (**B**), and DNA damage expressed as Olive Tail Moment arbitrary units (**C**) or AP sites (**D**). P-values are derived from paired samples *t*-test. Certain items on this figure have been adapted from Servier Medical Art by Servier (https://smart.servier.com—licensed under Creative Commons Attribution 3.0 Unported License; last accessed on 21 January 2022).

Next, we examined whether therapeutic intravenous iron administration affected the oxidative state of circulating mononuclear cells. For this purpose, three participants with iron deficiency anemia, who had no previous history of iron parenteric treatment, were given intravenous iron (ferric carboxymaltose, Ferrinject, Vifor, France) administration over 2 h (500 mg Fe(III) diluted in 250 mL of normal saline). Blood was collected before and immediately after the completion of iron administration and PBMCs were isolated using standard methods (Figure 2A). We observed that therapeutic iron administration indeed led to increased oxidative stress levels as depicted by a lower GSH/GSSG ratio (P = 0.01, Figure 2B). Iron administration also led to increased levels of DNA SSBs and/or DSBs as measured by alkaline comet assay (P = 0.002, Figure 2C) and an increased number of abasic sites (P = 0.009, Figure 2D).

### 3.3. Iron Deposition in the Hands in Early SSc

**Figure 3:**
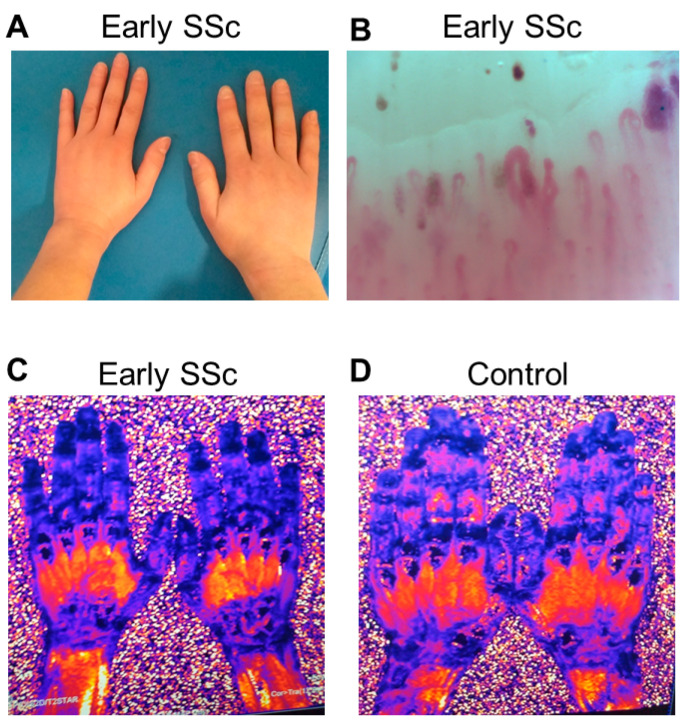
**Increased iron levels in the hands of a SSc patient.** A woman with early SSc and sclerodactyly (**A**) underwent capillaroscopy, which revealed multiple nailfold microhemorrhages (arrows) (**B**). Using a magnetic resonance imaging protocol for evaluation of iron deposition, depicted as black color in the T2(STAR) images, her fingers showed T2(STAR) values of 3 to 3.3 ms (**C**) denoting iron deposition compared to an apparently healthy control subject with higher values of 9.4 to 11 ms (**D**).

MRI of the hands (Figure 3A) was performed in a consenting 33-year-old woman with Raynaud’s and diffuse SSc of one year duration. Sclerodactyly was evident at clinical examination (Figure 3A), while microhemorrhages were evident in capillaroscopy (Figure 3B). The T2STAR image of her fingers showed extremely low values of 3 to 3.3 ms (Figure 3C) due to severe iron deposition compared to an apparently healthy control subject (SM, 9.4 to 11 ms) (Figure 3D).

## 4. Discussion

Previous studies in primary human fibroblasts and endothelial cells have shown that senescent cells accumulate intracellular iron [29]; in turn, the presence of intracellular labile iron (Fe(ΙΙ)) is a prerequisite for the development of oxidative stress-induced senescence and apoptosis [16]. Herein, we show that iron overload leads to oxidative DNA damage in both skin and synovial fibroblasts, suggesting that iron overload may be an additional source of the increased oxidative burden present in fibroblasts isolated from SSc patients [30,31]. Regarding the iron concentration used in our experiments (10–100 μΜ), previous studies have shown that iron concentration in human dermis under normal conditions is lower by up to 20-fold (0.5 μΜ) comparing to serum (10–35 μΜ) [32]. However, red blood cells contain extremely high amounts of iron (up to 2000-fold comparing to serum and almost 70% of the total body amount of iron) [33]. Therefore, repetitive/continuous red blood cell extravasation and lysis, as observed in SSc, could dramatically increase local iron concentration in the patients’ tissues.

Other studies have also shown that iron administration in vitro leads to DNA damage formation in PBMCs isolated from healthy individuals [27]. Herein we further show that the accumulation of DNA SSBs and DSBs in iron-treated PBMCs can be attributed to increased oxidative stress. As previously shown, the accumulation of DNA damage in PBMCs from patients with SSc associates with the extent of fibrosis in the skin and internal organs [28]. Moreover, herein, we show that therapeutic intravenous iron administration in humans also leads to increased oxidative stress and DNA damage accumulation in circulating lymphocytes and monocytes, which is in line with the existing literature [34,35,36,37]. Clearly, although the iron deposited in the tissues of SSc patients is unlikely to affect immune cells as it affects PBMCs when injected intravenously, these data suggest that what happens in the presence of iron in vitro also happens in vivo. As previously shown, repeated intravenous iron administration leads to increased protein oxidation, which is more pronounced in patients with a higher baseline inflammatory status, suggesting that iron may locally act as “fuel-on-fire” in conditions characterized by chronic inflammation [38]. Whether iron directly affects extracellular matrix production, profibrotic cytokine expression such as TGF-beta, myofibroblast differentiation, or EndoMT was not addressed herein and is currently under investigation.

MRI has been long established as an accurate, noninvasive method of detecting and quantifying iron overload [39], primarily in patients with thalassemia and repetitive blood transfusions. MRI has been shown to be clinically useful in quantifying iron overload in the liver [40,41], myocardium [41], bone marrow [42], and kidneys [43]. In addition, MRI is a noninvasive means of monitoring chelation treatment aiming at removing the excess iron [44]. Herein, in a case-report, we found increased iron levels by MRI in the hands of a patient with early SSc and capillaroscopic evidence of microhemorrhage, by more than threefold compared to a healthy volunteer. Since comparison of only one patient and one control cannot determine whether this result is unique in early SSc, we are currently conducting a systematic MRI study to examine tissue iron levels, including internal organs, in patients with diffuse and limited SSc of variable disease duration and severity. The possible correlation between the extent of nailfold hemorrhagic scores [45] and individual iron levels in the hands of these patients is also underway. Since capillaroscopic evidence of microhemorrhage, albeit to a far lesser extent [46], has been observed in patients with systemic lupus erythematosus and dermatomyositis, such patients will serve as disease controls.

**Figure 4:**
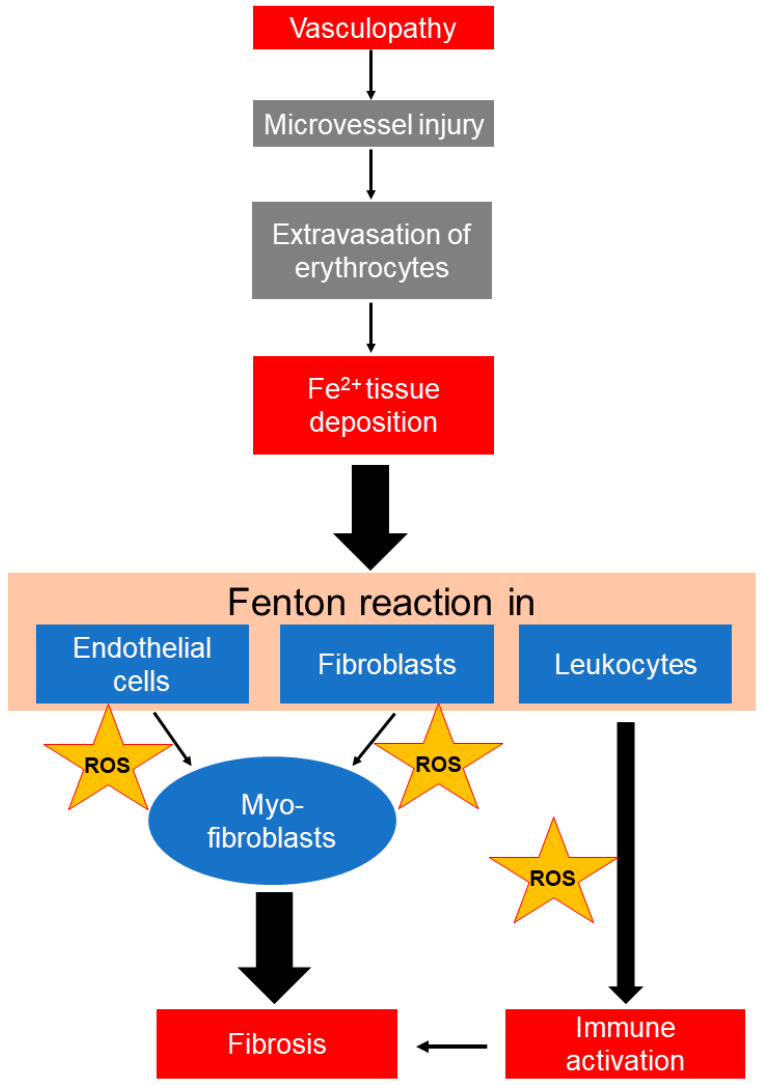
**Proposed mechanistic associations between vasculopathy, fibrosis, and immune activation in SSc.** Microvasculopathy observed in SSc may lead to repetitive/continuous hemorrhagic tissue deposition of iron, which, once taken up by resident cells, leads to the generation of extremely reactive hydroxyl radicals (^•^OH) through the Fenton reaction. Published data suggest that iron promotes endothelial-to-mesenchymal (myofibroblast) transition, fibroblast-to-myofibroblast transition associated with increased collagen production, and activation of immune cells, mainly through reactive oxygen species (ROS) formation.

Based on the above we propose (Figure 4) that microhemorrhagic iron deposition as a result of repetitive/continuous microvascular injury in SSc may further affect tissue resident cells, such as fibroblasts and endothelial cells which acquire a profibrotic phenotype, as well as activate tissue infiltrating leukocytes, which exert deleterious effects, including endothelial apoptosis [47]. Other published data support our hypothesis. For example, erythrocyte-derived tissue iron overload, i.e., after intracranial hemorrhage, has been shown to increase the oxidative burden of endothelial cells, leading to a more extensive secondary damage of surrounding tissues and worse clinical outcomes [48]. Of note, the administration of heme scavengers or iron chelation may limit secondary tissue damage [48,49]. Similarly, in vitro treatment of endothelial cells with iron leads to increased ROS production [24]. Iron overload may also induce EndoMT, while this phenotypic switch is attenuated by ROS scavengers [24].

The transformation of endothelial cells in profibrotic myofibroblasts also seems to be integral for SSc-related fibrosis [6,8,10,50], since cells undergoing EndoMT were detected in the skin vessels of SSc patients, as well as in animal models of SSc (bleomycin-induced fibrosis/urokinase-type plasminogen activator receptor (uPAR)-deficient mice) [6]. Similar to endothelial cells, the treatment of human lung fibroblasts with ferric ammonium citrate increased proinflammatory and extracellular matrix gene expression [23]. In line with this, mice with extensive iron accumulation due to defects in key iron transporters, display increased collagen deposition around small airways and a deterioration of lung function after methacholine administration [23]. On the other hand, in the model of bleomycin-induced fibrosis, increased iron deposition is detected in lung tissues at late stages coinciding with small airway fibrosis and a reduced diffusing capacity, while mice with pre-existing iron overload develop more pronounced lung fibrosis after bleomycin administration compared to wild-type mice [23]. Finally, as early studies had shown, iron chelating agents can inhibit the activation of mononuclear cells [51,52,53]. As more recently shown, iron leads to increased expression of CCL2, IL-6, IL-1, TNF, and iNOS in macrophages, which is reversible by chelating agents [54].

As a next step, it is tempting to hypothesize that targeting vascular leakage [55], or iron deposition per se through chelation, could limit oxidative stress and prevent profibrotic transformation of tissue resident cells in SSc. Indeed, in vitro studies have shown that treatment with the iron chelator deferoxamine prevents the oxidative stress-induced senescence of cells, while treatment with potent antioxidants such as ascorbic acid, tocopherol, or N-acetylcysteine did not (reviewed in [16]). Similarly, the administration of deferoxamine in bleomycin-treated mice significantly reduced BALF inflammatory infiltrates, fibrotic lung transformation, and functional lung decline [23,56].

To conclude, in the present preliminary study we report that: (1) in vitro treatment with iron induces oxidative stress and DNA damage in fibroblasts and blood mononuclear cells; (2) intravenous therapeutic administration of iron induces oxidative stress in blood mononuclear cells in vivo; (3) iron-induced oxidative stress is biologically relevant, as shown by the concomitant DNA damage accumulation; (4) increased iron deposition in the hands can be demonstrated by MRI in SSc. Based on these preliminary findings and on several lines of experimental evidence discussed above, we propose that microvasculopathy-related hemorrhagic tissue deposition of iron may contribute to fibrosis in the skin and internal organs in SSc. Thus, as depicted in Figure 4, erythrocyte-derived iron uptake by resident endothelial cells, fibroblasts, and infiltrating leukocytes may be, in parallel to the inflammatory process, an additional trigger of increased oxidative stress and damage in SSc, thus being an additional pathogenetic link between microvasculopathy and fibrosis, as well as a potential treatment target that deserves further study.

## Data Availability

Data are available by the corresponding author upon reasonable request.
